# Coexpression and coregulation analysis of time-series gene expression data in estrogen-induced breast cancer cell

**DOI:** 10.1186/1748-7188-8-9

**Published:** 2013-03-23

**Authors:** Anirban Bhar, Martin Haubrock, Anirban Mukhopadhyay, Ujjwal Maulik, Sanghamitra Bandyopadhyay, Edgar Wingender

**Affiliations:** 1 Institute of Bioinformatics, University Medical Center Goettingen, University of Goettingen, Goldschmidtstrasse 1, D-37077 Goettingen, Germany; 2 Department of Computer Science and Engineering, University of Kalyani, Kalyani-741235, India; 3 Department of Computer Science and Engineering, Jadavpur University, Kolkata-700032, India; 4 Machine Intelligence Unit, Indian Statistical Institute, Kolkata-700108, India

**Keywords:** Time series gene expression data, Tricluster, Mean-squared residue, Eigengene, Affirmation score, Gene ontology, KEGG pathway, TRANSFAC

## Abstract

**Background:**

Estrogen is a chemical messenger that has an influence on many breast cancers as it helps cells to grow and divide. These cancers are often known as estrogen responsive cancers in which estrogen receptor occupies the surface of the cells. The successful treatment of breast cancers requires understanding gene expression, identifying of tumor markers, acquiring knowledge of cellular pathways, etc. In this paper we introduce our proposed triclustering algorithm *δ*-TRIMAX that aims to find genes that are coexpressed over subset of samples across a subset of time points. Here we introduce a novel mean-squared residue for such 3D dataset. Our proposed algorithm yields triclusters that have a mean-squared residue score below a threshold *δ*.

**Results:**

We have applied our algorithm on one simulated dataset and one real-life dataset. The real-life dataset is a time-series dataset in estrogen induced breast cancer cell line. To establish the biological significance of genes belonging to resultant triclusters we have performed gene ontology, KEGG pathway and transcription factor binding site enrichment analysis. Additionally, we represent each resultant tricluster by computing its eigengene and verify whether its eigengene is also differentially expressed at early, middle and late estrogen responsive stages. We also identified hub-genes for each resultant triclusters and verified whether the hub-genes are found to be associated with breast cancer. Through our analysis *CCL2, CD47, NFIB, BRD4, HPGD, CSNK1E, NPC1L1, PTEN, PTPN2 and ADAM9* are identified as hub-genes which are already known to be associated with breast cancer. The other genes that have also been identified as hub-genes might be associated with breast cancer or estrogen responsive elements. The TFBS enrichment analysis also reveals that transcription factor *POU2F1* binds to the promoter region of *ESR1* that encodes estrogen receptor *α*. Transcription factor *E2F1* binds to the promoter regions of coexpressed genes *MCM7, ANAPC1 and WEE1*.

**Conclusions:**

Thus our integrative approach provides insights into breast cancer prognosis.

## Background

In the context of genomics research, the functional approach is based on the ability to analyze genome-wide patterns of gene expression and the mechanisms by which gene expression is coordinated. Microarray technology and other high-throughput methods are used to measure expression values of thousands of genes over different samples/experimental conditions. In recent years the microarray technology has been used to measure in a single experiment expression values of thousands of genes under a huge variety of experimental conditions across different time points. This kind of datasets can be referred to as time series microarray datasets. Because of the large data volume, computational methods are used to analyze such datasets. Clustering is one of the most common methods for identifying coexpressed genes [1]. This kind of analysis is facilitative for constructing gene regulatory networks in which single or groups of genes interact with other genes. Besides this, coexpression analysis also reveals information about some unknown genes that form a cluster with some known genes.

A clustering algorithm is used to group genes that are coexpressed over all conditions/samples or to group experimental conditions over all genes based on some similarity/dissimilarity metric. However clustering may fail to find the group of genes that are similarly expressed over a subset of samples/experimental conditions i.e. clustering algorithms are unable to find such local patterns in the gene expression dataset. To deal with that problem, biclustering algorithms are used. A bicluster can be defined as a subset of genes that are coexpressed over a subset of samples/experimental conditions. The first biclustering algorithm that was used to analyse gene expression datasets was proposed by Cheng and Church and they used a greedy search heuristic approach to retrieve largest possible bicluster having mean squared residue (MSR) under a predefined threshold value *δ* (*δ*-bicluster) [2]. But nowadays, biologists are eager to analyze 3D microarray dataset to answer the question: “*Which genes are coexpressed under which subset of experimental conditions/samples across which subset of time points*?” Biclustering is not able to deal with such 3D datasets. So, in this case we need some other clustering technique that can mine 3D datasets. Hence the term *Triclustering* has been defined and a tricluster can be delineated as a subset of genes that are similarly expressed across a subset of experimental conditions/samples over a subset of time points. Zhao and Zaki proposed a triclustering algorithm *TRICLUSTER* that is based on graph-based approach. They defined coherence of a tricluster as $\frac{\text{max}(e_{\text{ib}}/e_{\text{ia}},e_{\text{jb}}/e_{\text{ja}})}{\text{min}(e_{\text{ib}}/e_{\text{ia}},e_{\text{jb}}/e_{\text{ja}})}-1$, where *e*_*ia*_,*e*_*ib*_ denote the expression values of two columns a and b respectively for a row i. A tricluster is valid if it has a ratio below a maximum ratio threshold *ϵ*[3].

Here we introduce an efficient triclustering algorithm *δ-TRIMAX*[4] that aims to cope with noisy 3D gene expression dataset and is less sensitive to input parameters. The normalization method does not influence the performance of our algorithm, as it produces the same results for both normalized and raw datasets. Here we propose a novel extension of MSR [2] for 3D gene expression data and use a greedy search heuristic approach to retrieve triclusters, having MSR values below a threshold *δ*. Hence the triclusters can be defined as *δ*-tricluster.

In this work we have applied our proposed *δ*-TRIMAX algorithm on a time-series gene expression data in estrogen induced breast cancer cell. Estrogen, a chemical messenger plays an instrumental role in normal sexual development, regulating woman’s menstrual cycles and normal development of the breast. Estrogen is also needed for heart and healthy bones. As estrogen plays vital role in stimulating breast cell division, has an effect on other hormones implicated in breast cell division and provides support to the growth of estrogen-responsive tumors, it may be involved in risk for breast cancer [5]. Though since last decade, some research has been done to decipher some unknown questions on breast cancer risk, still some questions such as involvement of genes in breast cancer risk etc. remain unanswered. Here our coexpression analysis reveals some genes that have already been found to be associated with estrogen induced breast cancer and some other genes that might play an important role in this context. Additionally, our coregulation analysis brings out some important information such as which transcription factor binds the promoter regions of genes and play an important role in this context.

In section 2, we have described our proposed triclustering algorithm in detail. Section 3 shows results of our algorithm using one artificial dataset and one real-life dataset. In section 4, we conclude our work.

## Methods

### Definitions

#### 

**Definition 1 (Time Series Microarray Gene Expression Dataset).** We can model a *time series microarray gene expression dataset* (D) as a G × C × T matrix and each element of D (d _*ijk*_) corresponds to the expression value of gene i over jth sample/experimental condition across time point k, where i ∈ (g_1_,g_2_,...,g _*G*_), j ∈ (c_1_,c_2_,...,c _*C*_) and k ∈ (t_1_,t_2_,...,t _*T*_).

#### 

**Definition 2** (Tricluster). A *tricluster* is defined as a submatrix M(I,J,K) = [m _*ijk*_], where i ∈ I, j ∈ J and k ∈ K. The submatrix M represents a subset of genes (I) that are coexpressed over a subset of conditions (J) across a subset of time points (K).

#### 

**Definition 3** (Perfect Shifting Tricluster). A Tricluster M(I,J,K) = m _*ijk*_, where i ∈ I, j ∈ J and k ∈ K, is called a *perfect shifting tricluster* if each element of the submatrix M is represented as: m _*ijk*_ = *Γ* + *α*_*i*_ + *β*_*j*_ + *η*_*k*_, where *Γ* is a constant value for the tricluster, *α*_*i*_, *β*_*j*_ and *η*_*k*_ are shifting factors of ith gene, jth samples/experimental condition and kth time point, respectively. As the noise is present in microarray datasets, the deviation from actual value and expected value of each element in the dataset also exists. For this deviation, every tricluster is not a perfect one.

Cheng and Church proposed an algorithm for retrieving large and maximal biclusters that have mean squared residue score (MSR) below a threshold *δ* in 2D microarray gene expression dataset. They also showed that MSR of a perfect *δ*-bicluster and perfect shifting bicluster is zero (**S** = *δ* = 0) [2,6]. Now extending this idea, here we present a novel definition of Mean Squared Residue score for 3D microarray gene expression datasets. The MSR (**S**) of a perfect shifting tricluster becomes also zero, where each element *m*_*ijk*_ = *Γ* + *α*_*i*_ + *β*_*j*_ + *η*_*k*_. For delineating new MSR score (**S**), at first we need to define the residue score:

Let the mean of *i*th gene (m _*ijk*_): $m_{\text{iJK}}=\frac{1}{\left|J||K\right|}\sum_{j\in J,k\in K}\;m_{\text{ijk}}$, the mean of *j*th sample/experimental condition (m _*ijk*_): $m_{\text{IjK}}=\frac{1}{\left|I||K\right|}\sum_{i\in I,k\in K}m_{\text{ijk}}$, the mean of *k*th time point (m _*ijk*_): $m_{\text{IJk}}=\frac{1}{\left|I||J\right|}\sum_{i\in I,j\in J}m_{\text{ijk}}$, and the mean of tricluster (m _*ijk*_): $m_{\text{IJK}}=\frac{1}{\left|I||J||K\right|}\sum_{i\in I,j\in J,k\in K}m_{\text{ijk}}$. Now the mean of the tricluster can be considered as the value of constant i.e. *Γ*=*m*_*IJK*_. We can define the shifting factor for the ith gene (*α*_*i*_) as the difference between m _*ijk*_ and m _*ijk*_ i.e. *α*_*i*_=*m*_*iJK*_-*m*_*IJK*_. Similarly, we can define shifting factor for the jth condition (*β*_*j*_) as *β*_*j*_=*m*_*IjK*_-*m*_*IJK*_ and shifting factor for the kth time point (*η*_*k*_) can be defined as *η*_*k*_=*m*_*IJk*_-*m*_*IJK*_. Hence we can define each element of a perfect shifting tricluster as *m*_*ijk*_=*Γ*+*α*_*i*_+*β*_*j*_+*η*_*k*_=*m*_*IJK*_+(*m*_*iJK*_-*m*_*IJK*_)+(*m*_*IjK*_-*m*_*IJK*_)+(*m*_*IJk*_-*m*_*IJK*_)=(*m*_*iJK*_+*m*_*IjK*_+*m*_*IJk*_-2*m*_*IJK*_). But usually noise is evident in microarray gene expression dataset. Therefore to evaluate the difference between the actual value of an element (m _*ijk*_) and its expected value, obtained from above equation, the term “*residue*” can be used [6]. Thus the residue of a tricluster (r _*ijk*_) can be defined as follows: *r*_*ijk*_=*m*_*ijk*_-(*m*_*iJK*_+*m*_*IjK*_+*m*_*IJk*_-2*m*_*IJK*_)=(*m*_*ijk*_-*m*_*iJK*_-*m*_*IjK*_-*m*_*IJk*_+2*m*_*IJK*_).

#### 

**Definition** 4 (Mean Squared Residue). We define the term Mean Squared Residue MSR(I,J,K) or **S** of a tricluster M(I,J,K) to estimate the quality of a tricluster i.e. the level of coherence among the elements of a tricluster as follows:

(1) $$\begin{array}{ll} \;\text{S} & =\frac{1}{\left|I||J||K\right|}\underset{i\in I,j\in J,k\in K}{\sum }r_{\text{ijk}}^{2}\;\\ & =\frac{1}{\left|I||J||K\right|}\underset{i\in I,j\in J,k\in K}{\sum }\;\;\;{\left(m_{\text{ijk}}-m_{\text{iJK}}-m_{\text{IjK}}-m_{\text{IJk}}+2m_{\text{IJK}}\right)}^{2}.\; \end{array}$$

Lower residue score represents larger coherence and better quality of a tricluster.

### Proposed method

*δ-TRIMAX* aims to find largest and maximal triclusters in a 3D microarray gene expression dataset. It is an extension of Cheng and Church biclustering algorithm [2] that deals with 2-D microarray datasets. In contrast, our algorithm is capable to mine 3D gene expression dataset. There is always a submatrix in an expression dataset that has a perfect MSR(I,J,K) or **S** score i.e. **S** = 0 and this submatrix is each element of the dataset. But as mentioned above, our algorithm finds maximal triclusters having **S** score under a threshold *δ*, hence we have used a greedy heuristic approach to find triclusters. Our algorithm therefore starts with the entire dataset containing all genes, all samples/experimental conditions and all time points.

### Algorithm 1 (*δ*-TRIMAX)

**Input.** D, a matrix that represents 3D microarray gene expression dataset, *λ*> 1, an input parameter for multiple node deletion algorithm, *δ*≥ 0, maximum allowable MSR score.

**Output.** All possible *δ*-triclusters.

**Initialization.** Missing elements in D ← random numbers, D’ ← D

Repeat

a. D’_1_ ← Results of Algorithm 2 on D’ using *delta* and *λ*. If the no. of genes (conditions/samples and/or no. of time points) is 50 (This value can be choosen experimentally. Large value increases the execution time of the algorithm as it then executes more number of iterations.), then do not apply Algorithm 2 on genes (conditions/samples and/or time points).

b. D’_2_ ← Results of Algorithm 3 on D’_1_ using*δ*.

c. D’_3_ ← Results of Algorithm 4 on D’_2_.

d. Return D’_3_ and replace the elements that exist in D’ and D’_3_ with random numbers.

**Until**(No gene is found for *δ*-tricluster)

Initially, our algorithm removes genes or conditions or time points from the dataset to accomplish largest diminishing of score **S**; this step is described in the following section in which a node corresponds to a gene or experimental condition or time point in the 3D microarray gene expression dataset.

### Algorithm 2 (Multiple node deletion)

**Input.** D, a matrix of real numbers that represents 3D microarray gene expression dataset; *δ*≥ 0, maximum allowable MSR threshold, *λ*> 1, threshold for multiple node deletion. The value of *λ* has been set experimentally to optimize the speed and performance (to avoid falling into local optimum) of the algorithm.

**Output.** M _*ijk*_, a *δ*-tricluster, consisting of a subset(I) of genes, a subset(J) of samples/experimental conditions and a subset of time points, having MSR score (**S**) less than or equal to *δ*.

**Initialization.** I ←{set of all genes }, J ←{set of all experimental conditions/ samples } and K ←{set of all time points } and to M(I,J,K) ← D

Repeat

Calculate m _*ijk*_, ∀ i ∈ I; m _*ijk*_, ∀ j ∈ J; m _*ijk*_, ∀ k ∈ K; m _*ijk*_ and **S**.

**If S** ≤*δ* return M(I,J,K)

Else

Delete genes i ∈ I that satisfy the following inequality

$$\frac{1}{\left|J||K\right|}\Sigma_{j\in J,k\in K}{\left(m_{\text{ijk}}-m_{\text{iJK}}-m_{\text{IjK}}-m_{\text{IJk}}+2m_{\text{IJK}}\right)}^{2}>\lambda \text{S}$$

Recalculate m _*ijk*_, ∀ i ∈ I; m _*ijk*_, ∀ j ∈ J; m _*ijk*_, ∀ k ∈ K; m _*ijk*_ and **S**

Delete samples/experimental conditions j ∈ J that satisfy the following inequality

$$\frac{1}{\left|I||K\right|}\Sigma_{i\in I,k\in K}{\left(m_{\text{ijk}}-m_{\text{iJK}}-m_{\text{IjK}}-m_{\text{IJk}}+2m_{\text{IJK}}\right)}^{2}>\lambda \text{S}$$

Recalculate m _*ijk*_, ∀ i ∈ I; m _*ijk*_, ∀ j ∈ J; m _*ijk*_, ∀ k ∈ K; m _*ijk*_ and **S**

Delete time points k ∈ K that satisfy the following inequality

$$\frac{1}{\left|I||J\right|}\Sigma_{i\in I,j\in J}{\left(m_{\text{ijk}}-m_{\text{iJK}}-m_{\text{IjK}}-m_{\text{IJk}}+2m_{\text{IJK}}\right)}^{2}>\lambda \text{S}$$

End if

**Until**(There is no change in I, J and/or K)

The complexity of this algorithm is O(max(m,n,p)) where m, n and p are the number of genes, samples and time points in the 3D microarray dataset.

In the second step, we delete one node at each iteration from the resultant submatrix, produced by Algorithm 2, until the score **S** of the resultant submatrix is less than or equal to *δ*. This step results in a *δ*-tricluster.

### Algorithm 3 (Single node deletion)

**Input.** D, a matrix of real numbers that represents 3D microarray gene expression dataset; *δ*≥ 0, maximum allowable MSR threshold.

**Output.** M _*ijk*_, a *δ*-tricluster, consisting of a subset(I) of genes, a subset(J) of samples/experimental conditions and a subset of time points, having MSR score (**S**) less than or equal to *δ*.

**Initialization.**I ←{set of all genes in D }, J ←{set of experimental conditions/samples in D } and K ←{set of time points in D } and to M(I,J,K) ← D

Calculate m _*ijk*_, ∀ i ∈ I; m _*ijk*_, ∀ j ∈ J; m _*ijk*_, ∀ k ∈ K; m _*ijk*_ and **S**.

**While S** >*δ*

Detect gene i ∈ I that has the highest score

$$\mu (i)=\frac{1}{\left|J||K\right|}\Sigma_{j\in J,k\in K}{\left(m_{\text{ijk}}-m_{\text{iJK}}-m_{\text{IjK}}-m_{\text{IJk}}+2m_{\text{IJK}}\right)}^{2}$$

Detect sample/experimental condition j ∈ J that has the highest score

$$\mu (j)=\frac{1}{\left|I||K\right|}\Sigma_{i\in I,k\in K}{\left(m_{\text{ijk}}-m_{\text{iJK}}-m_{\text{IjK}}-m_{\text{IJk}}+2m_{\text{IJK}}\right)}^{2}$$

Detect time point k ∈ K that has the highest score

$$\mu (k)=\frac{1}{\left|I||J\right|}\Sigma_{i\in I,j\in J}{\left(m_{\text{ijk}}-m_{\text{iJK}}-m_{\text{IjK}}-m_{\text{IJk}}+2m_{\text{IJK}}\right)}^{2}$$

Delete gene or sample/experimental condition or time point that has highest *μ* score and modify I or J or K. Recalculate m _*ijk*_, ∀ i ∈ I; m _*ijk*_, ∀ j ∈ J; m _*ijk*_, ∀ k ∈ K; m _*ijk*_ and **S**.

End while

Return M(I,J,K)

The complexity of first and second steps is O(mnp) as those will iterate (m+n+p) times. The complexity of selection of best genes, samples and time points is O(log m + log n + log p). So it is suggested to use algorithm II before algorithm 3.

As the goal of our algorithm is to find maximal triclusters, having MSR score (**S**) below the threshold *δ*, the resultant tricluster M(I,J,K) may not be the largest one. That means some genes and/or experimental conditions/samples and/or time points may be added to the resultant tricluster T produced by node deletion algorithm, so that the MSR score of new tricluster T’ produced after node addition does not exceed the MSR score of T. Now the third step of our algorithm is described below.

### Algorithm 4 (Node addition)

**Input.** D, a matrix of real numbers that represents *δ*-tricluster, having a subset of genes (I), a subset of experimental conditions/samples (J) and a subset of time points (K).

**Output.** M${}_{I^{\prime }J^{\prime }K^{\prime }}$, a *δ*-tricluster, consisting of a subset of genes (I’), a subset of samples/experimental conditions (J’) and a subset of time points (K’), such that I ⊂ I ^′^, J ⊂ J ^′^, K ⊂ K’ and MSR(I ^′^,J ^′^,K’) ≤ MSR of D.

**Initialization.** M(I,J,K) ← D

Repeat

Calculate m _*ijk*_, ∀ i; m _*ijk*_, ∀ j; m _*ijk*_, ∀ k; m _*ijk*_ and **S**. Add genes i ∉ I that satisfy the following inequality

$$\frac{1}{\left|J||K\right|}\Sigma_{j\in J,k\in K}{\left(m_{\text{ijk}}-m_{\text{iJK}}-m_{\text{IjK}}-m_{\text{IJk}}+2m_{\text{IJK}}\right)}^{2}\leq \text{S}$$

Recalculate m _*ijk*_, ∀ j; m _*ijk*_, ∀; m _*ijk*_ and **S**

Add samples/experimental conditions j ∉ J that satisfy the following inequality

$$\frac{1}{\left|I||K\right|}\Sigma_{i\in I,k\in K}{\left(m_{\text{ijk}}-m_{\text{iJK}}-m_{\text{IjK}}-m_{\text{IJk}}+2m_{\text{IJK}}\right)}^{2}\leq \text{S}$$

Recalculate m _*ijk*_, ∀ i; m _*ijk*_, ∀ k; m _*ijk*_ and **S**

Add time points k ∉ K that satisfy the following inequality

$$\frac{1}{\left|I||J\right|}\Sigma_{i\in I,j\in J}{\left(m_{\text{ijk}}-m_{\text{iJK}}-m_{\text{IjK}}-m_{\text{IJk}}+2m_{\text{IJK}}\right)}^{2}\leq \text{S}$$

**Until**(There is no change in I, J and/or K)

I’ ← I, J’ ← J, K’ ← K Return I’, J’, K’

The complexity of this algorithm is O(mnp) as each step iterates (m+n+p) times.

### Tricluster eigengene

To find tricluster eigengene we applied singular value decomposition method (SVD) on the expression data of each tricluster [7]. For instance, $X_{g\times (c*t)}^{i}$ represents the expression matrix of ith tricluster, where g, c and t represent the number of genes, samples and time points of ith tricluster. Now we apply SVD on the data matrix (normalized to mean=0 and variance=1). Now, the SVD of ith tricluster can be represented as,

(2) $$X^{i}=\text{UD}V^{T},$$

where U and V are the orthogonal matrices. *U*^*i*^ is a g ∗ (c ∗ t) matrix with orthonormal columns, *V*^*i*^ is a (c ∗ t) × (c ∗ t) orthogonal matrix and *D*^*i*^ is (c ∗ t) × (c ∗ t) diagonal matrix of singular values.

Assuming that singular values in matrix *D*^*i*^ are arranged in non-decreasing order, we can represent eigengene of ith tricluster by the first column of matrix *V*^*i*^, i.e.

(3) $$E^{i}=V_{1}^{i},$$

## Results and discussion

### Results on simulated dataset

We have produced one simulated dataset SMD of size 2000 × 30 × 30. At first we have implanted three perfect shifting triclusters of size 100 × 6 × 6, 80 × 6 × 6 and 60 × 5 × 5 into the dataset SMD and then implanted three noisy shifting triclusters of the same size mentioned before into it. To estimate the degree of similarity between the implanted and obtained triclusters, we define *affirmation score* in the same way as Prelic et. al. defined for two sets of biclusters [6,8]. So, overall average affirmation score of T_1_ with respect to T_2_ is as follows, where (SM${}_{G}^{*}$(T_1_, T_2_)) is the average gene affirmation score, (SM${}_{C}^{*}$(T_1_, T_2_)) is the average sample affirmation score and (SM${}_{K}^{*}$(T_1_, T_2_)) is the average time point affirmation score of T_1_ with respect to T_2_:

(4) $$\;\begin{array}{c} SM^{*}(T_{1},T_{2})\\ \;=\sqrt{\left(SM_{G}^{*}(T_{1},T_{2})\;\times \;SM_{C}^{*}(T_{1},T_{2})\times SM_{T}^{*}(T_{1},T_{2})\right)} \end{array}$$

Suppose, we have two sets of triclusters T _*im*_ and T _*res*_ where T _*im*_ represents the set of implanted triclusters and T _*res*_ corresponds to the set of triclusters retrieved by any triclustering algorithm. Hence SM ^∗^(*T*_*im*_,*T*_*res*_) denotes how well the triclustering algorithm finds the true triclusters that have been implanted into the dataset. This score varies from 0 to 1 (if T _*im*_ = T _*res*_).

**Figure 1:**
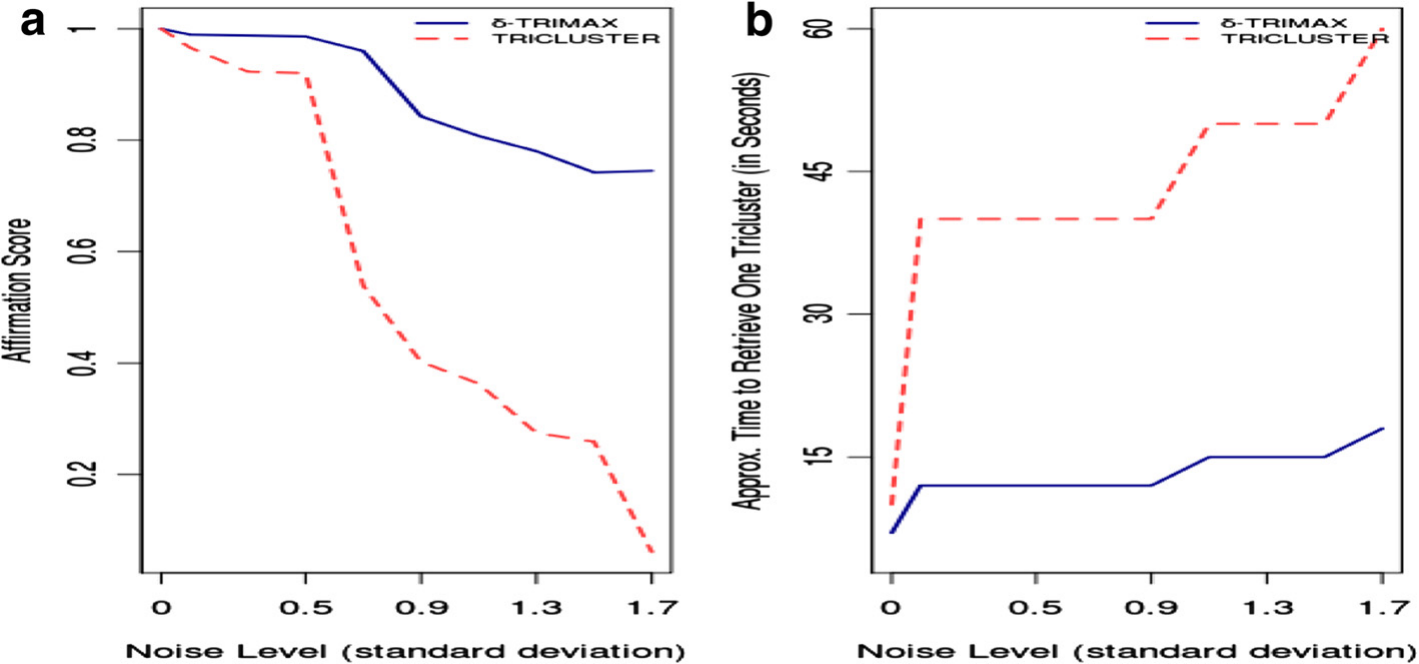
**Comparison in terms of Affirmation Scores.a.** Comparison of Affirmation scores produced by -TRIMAX and TRICLUSTER algorithm. **b.** Comparison of running time of -TRIMAX and TRICLUSTER algorithm on the synthetic dataset.

For the dataset containing perfect shifing triclusters, we have assigned 0.35 and 1.0005 to the parameters *δ* and *λ*, respectively. The value of *δ* varies from one dataset to another dataset. Then we have added noisy triclusters having different levels i.e. standard deviations (*σ* = 0.1, 0.3, 0.5, 0.7, 0.9, 1.1, 1.3, 1.5, 1.7). To have an idea about the *δ* value, we have first clustered the genes over all time points and then the time points over the subset of genes for each gene cluster in each sample plane using the K-means algorithm. Then we have computed the MSR value(S) of the submatrix, considering a randomly selected sample plane, gene and time-pont cluster for 100 times. Then we have taken the lowest value as the value of *δ*. For these noisy datasets, we have assigned 3.75 and 1.004 to the parameters *δ* and *λ*, respectively. In Figure 1 we have compared the performance of our algorithm with that of the *TRICLUSTER* algorithm [3] in terms of affirmation score using the artificial dataset. Our *δ*-TRIMAX algorithm performs better than *TRICLUSTER* algorithm for the noisy dataset. For perfect additive triclusters, performances of both these algorithms are comparable with each other.

### Results on real-life dataset

#### Datasets for genome-wide analysis of estrogen receptor binding sites

**Figure 2:**
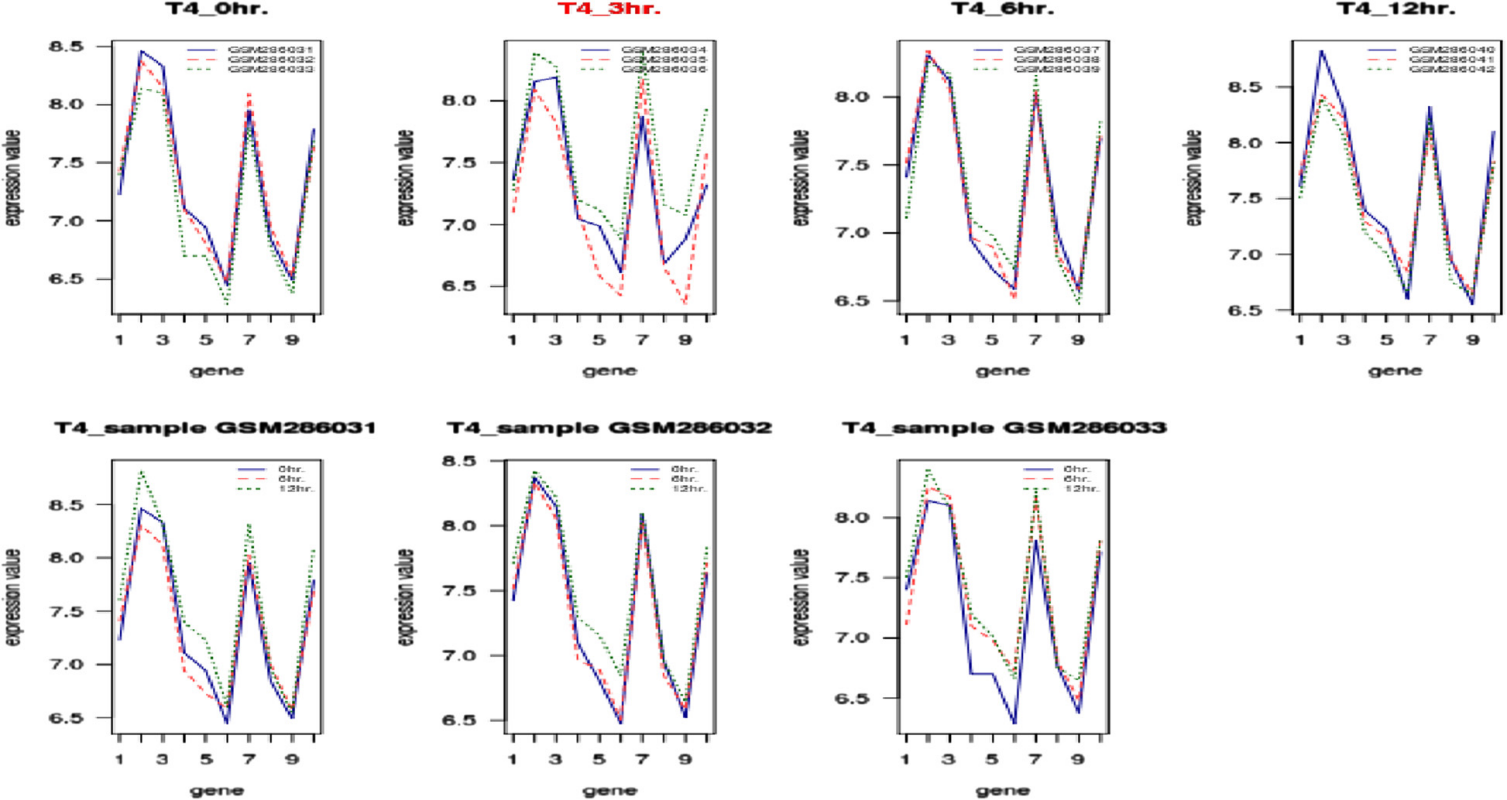
**Expression Profiles.** Figures in first row show the expression profiles of genes *ESR1, HOXA11, FAM71A, SPEF2, IFIH1, FPR2, SPAG9, NCF4, ADAM3A, CCNYL1* respectively of tricluster 4 over all samples; The red-colored time point (3 hrs.) is not a member of this tricluster. The figures in second row show the expression profiles of the same genes across 0, 6 and 12 hours.

This dataset contains 54675 affymetrix probe-set ids, 3 biological replicates and 4 time points. In this experiment MCF7 cells are stimulated with 100 nm estrogen for 0, 3, 6 and 12 hours and the experiments are performed in triplicate. This dataset is publicly available at Gene Expression Omnibus (GEO) (dataset id- GSE 11324). It was used for discovering of cis-regulatory sites in previously uninvestigated regions and cooperating transcription factors underlying estrogen signaling in breast cancer [9]. We assign 0.012382 and 1.2 to *δ* and *λ* respectively. In this case our algorithm results in 115 triclusters. From Figure 2, we observe that the genes in tricluster 4 have similar expression profiles over all three samples across 0, 6 and 12 hours but not at 3 hour.

To compare the performance of our proposed algorithm with TRICLUSER algorithm on real-life dataset, we have used three validation indexes.

#### Coverage

Coverage for any triclustering algorithm can be delineated as

(5) $$\text{Coverage}=\left(\frac{g_{\text{alg}}\times c_{\text{alg}}\times t_{\text{alg}}}{G\times C\times T}\right)\times 100,$$

where *g*_*alg*_, *c*_*alg*_ and *t*_*alg*_ denote total number of genes, experimental samples and time points retrieved by the triclustering algorithm. G, C and T represent number of all genes, experimental samples and time points in the dataset.

#### Triclustering Quality Index (TQI)

We can elucidate Triclusering Quality Index of a tricluster by equation 4.

(6) $$\text{TQI}=\frac{\text{MS}R_{i}}{\text{Volum}e_{i}},$$

where *MSR*_*i*_ and *Volume*_*i*_ represent mean-squared residue and volume of ith tricluster. Lower TQI score represents better quality of tricluster.

#### Statistical Difference from Background (SDB)

Here we have introduced another quality measurement, termed as Statistical Differences from Background (SDB) [10] as

(7) $$\text{SDB}=\frac{1}{n}\overset{n}{\underset{i=1}{\sum }}\frac{\text{MS}R_{i}}{\frac{1}{r}\sum_{j=1}^{r}\text{RMS}R_{j}-\text{MSRi}},$$

where n is the total number of triclusters extracted by the algorithm. *MSR*_*i*_ represents mean squared residue of ith tricluster retrieved by the algorithm and *RMSR*_*j*_ represents mean squared residue of jth random tricluster having the same number of genes, experimental samples and time points as that of ith resultant tricluster. Here higher value of the denominator denotes better quality of the resultant tricluster. Hence, lower SDB score signifies better performance of the algorithm. Table 1 shows the comparison between proposed *δ*-TRIMAX algorithm and TRICLUSTER algorithm in terms of coverage, SDB and TQI score.

**Table 1:** **Comparison between *δ *-TRIMAX and TRICLUSTER algorithm using coverage, Statistical Difference of from Background (SDB) and Triclustering Quality Index (TQI)**

**Algorithm**	**Coverage**	**SDB**	**Average TQI**
*δ*-TRIMAX	93.7412	0.4670856	3.082684e-05
TRICLUSTER	72.34019	0.4775341	3.348486e-05

### Biological significance

We have established the biological significance of genes belonging to each resultant tricluster by performing (a) Gene Ontology (GO) and KEGG pathway enrichment analysis, (b) cogitating each tricluster with different estrogen-responsive stages (early (3 hour), middle (6 hour) and late (12 hour)), (c) identifying hub genes of each tricluster and (d) Transcription Factor Binding Site (TFBS) enrichment analysis.

#### GO and KEGG pathway enrichment analysis

We have used *GOStats* package [11] in R to perform GO and KEGG pathway enrichment analysis for establishing biological significance of genes belonging to each tricluster. We have adjusted the p-values using Benjamini-Hochberg FDR method [12] and considered those terms as significant ones that have a p-value below a threshold of 0.05. The smaller p-value represents higher significance level. We have found statistically enriched GO terms for genes belonging to each tricluster. We have compared the performance of our proposed *δ*-TRIMAX algorithm with that of TRICLUSTER algorithm on real-life dataset. For comparison of the performances we have considered GO Biological Processes (GOBP) and KEGG pathway terms that have already been reported to play an important role in estrogen induced breast cancer cell. Table 2 shows the comparison between *δ*-TRIMAX and TRICLUSTER algorithm in terms corrected p-values of GOBP and KEGG pathway terms *cell adhesion* and *Wnt signaling pathway* that are observed to be associated with estrogen induced breast cancer [13,14], respectively.

**Table 2:** **Comparison between *δ *-TRIMAX and TRICLUSTER algorithm in terms of p-values of GO and KEGG pathway term enrichment analysis**

**Algorithm**	**GOBP term**	**KEGG pathway terms**
*δ*-TRIMAX	GO:0007155: cell adhesion	KEGG:04310: Wnt signaling
	(4.31e-08)	pathway (0.011)
TRICLUSTER	GO:0007155: cell adhesion	KEGG:04310: Wnt signaling
	(0.00022)	pathway (0.03)

#### Association of triclusters with different stages of response to estrogen stimulus

To cogitate each tricluster with different estrogen responsive stages of the experiment, we represent each tricluster by eigengene. Then we have examined whether the eigengene of each tricluster is differentially expressed at early, middle and late estrogen responsive stages using Limma package in R [15] (FDR-BH corrected p-value cutoff 0.05). If eigengene of one tricluster is found to be differentially expressed at any possible responsive stages, then the genes having highly correlated expression profiles with that of eigengene can also be considered to be significantly expressed at the same stages. In total our algorithm results in 115 triclusters. Eigengene of tricluster 7 has been found to be differentially expressed between 0 hour - 6 hours, 0 hour - 12 hours, 3 hours - 12 hours and 6 hours - 12 hours. 429 genes among 505 genes are found to be differentially expressed in this tricluster. KEGG pathway term *mTOR signaling pathway* is observed to be meliorated in this tricluster and has been reported to be associated with estrogen induced breast cancer cell [16]. Genes *PIK3CA, PRKAA1, RPS6, ULK2* participate in that pathway. The genes belonging to tricluster 50 are coexpressed over all samples across 0, 6 and 12 hours. The eigengene of tricluster 50 has been observed to be differentially expressed between 0 hour - 12 hours and 6 hours - 12 hours. 96% of the genes belonging to this tricluster are found to be differentially expressed. The genes in this tricluster are meliorated with the KEGG pathway term *ubiquitin mediated proteolysis* (*UBE2K, CUL4B, PIAS1, CDC23*). It has been reported in a previous study that there is crosstalk between ER *α* and targets of ER *α* for ubiquitin mediated proteolysis [17]. In tricluster 71 time points 3, 6 and 12 hours are present in that tricluster and the eigengene is significantly expressed between 3 hours and 12 hours. 44 genes out of 52 genes in this tricluster are significantly expressed between 3 and 12 hours. Genes belonging to this tricluster are also enriched with the KEGG pathway term *ubiquitin mediated proteolysis* (*UBA6, BIRC6, ANAPC1, CUL5*). Genes belonging to tricluster 48 are coexpressed across 0, 3 and 12 hours. Eigengene of tricluster 48 is significantly expressed between 0 and 12 hours, 3 and 12 hours. The KEGG pathway term *TGF-beta signaling pathway* is meliorated in this tricluster and the crosstalk between TGF-beta signaling pathway and ER *α* has been reported in a previous study [18]. Genes *SKP1, BMPR2* are found to play a role in the enriched pathway. Eigengene of tricluster 95 is significantly upregulated between 0 and 12 hours. 60% of all genes belonging to this tricluster are differentially up regulated at late responsive stage. The genes in this tricluster has been found to be coexpressed across 0 hour, 12 hours over all samples. The KEGG pathway term *apoptosis* (*XIAP, IRAK4, CASP6*) is observed to be meliorated in this tricluster and it has been found in a recent study that apoptosis can be induced by estrogen in estrogen deprivation-resistant breast cancer cell [19]. The genes of that tricluster 75 have been observed to be coexpressed over all samples across 3 and 12 hours. The eigengene of tricluster 75 is differentially expressed between 3 hours and 12 hours. 39 genes among 64 genes are significantly expressed between 3 and 12 hours. In this case we have observed enrichment for KEGG pathway terms *ErbB signaling pathway* that is found to be associated with estrogen induced breast cancer cell [20]. The coexpressed genes *NCK1, SOS2* in this tricluster participate in that pathway.

#### Identification and roles of hub genes

To identify hub genes of each tricluster, we have computed tricluster membership of each gene by calculating Pearson correlation coefficient between each gene and the eigengene of that tricluster. We have considered the top fifteen genes as hub genes having highest correlation coefficient with the eigengene of that tricluster. For tricluster 1, we have identified *NPC1L1, TMEM161B-AS1, POU5F1P3, POU5F1P4, POU5F1B, CCL2* as hub-genes that are coexpressed over all-time points. It has been observed in a previous study that high doses of estrogen augment intestinal cholesterol absorption attributable in part to an up-regulated expression of *NPC1L1* which is known as intestinal sterol influx transporter [21]. *CCL2* is found to play an important role in mediating cross-talk between cancer cells and stromal fibroblasts in breast cancer cells [22]. *DNAJC3-AS1, ITSN2, TRPC1, CD47, ZNF286A, TSC22D2, PHF17, ZNF286B, TMEM67, NFIB, JKAMP, DENND4A, HPGD* are identified as hub-genes that are coexpressed over all samples and 0, 3, 6 and 12 hours in tricluster 7. *NFIB* has been reported as a potential target of ER negative breast cancers [23]. Transient receptor potential cation channel (*TRPC1*) is known to play an important role in breast cancer [24]. *CD47* has been found to intervene killing of breast cancer cells [25]. *HPGD* plays important role in epithelial-mesenchymal transition and migration in breast cancer cells [26]. In tricluster 4, the genes are coexpressed over 0, 6 and 12 hours and *IGKV1-13, FAM69C, SGCD, CSNK1E, TRMU, CRYBA2, IGKV1D-13, IGSF11, PACS1, IQCK* are identified as hub-genes. *CSNK1E* has been observed to play an important role proliferation of breast cancer cells and act as a regulator of activated -catenin driven transcription [27]. For tricluster 13 we have identified *ESYT3, SERINC2, LRRC14, ALDH4A1, RPL10, BRD4, DEC1, ZFP30, TCP11L2, ALDOA* as hub-genes. The gene for Aldolase A (*ALDOA*) plays an instrumental role in hypoxia which is a feature of solid tumors in breast cancer [28]. Besides this *BRD4* known as Bromodomain 4 is found to be associated with breast cancer progression [29]. Genes *PFKFB1, TAF1, PIKFYVE, MEMO1P1, KIF1B, PHF20L1, ARHGAP24, TSC22D1, AK7, DPY30, MEMO1, PTEN, ADAM9, PTPN2, MTSS1L* are found as hubgenes of tricluster 95. *PTEN* is known to be a tumor suppressor gene in breast cancer [30,31]. *PTPN2, ADAM9* have been reported to be associated with breast cancer in previous studies [32,33]. *PIKFYVE* has been found to intervene epidermal growth factor receptor that is associated with human breast cancers [34]. In case of tricluster 42, *ANTXR2, RHBDL2, GSTCD, DENND1B, KLC3, PREP, NOS1, STOML3, CDK5R1, CLEC7A, HGD, FOXC1, MSRB3, TEX34, SLC36A1* are appeared as hub-genes that are coexpressed over all samples and across 0, 12 hours. In a recent work, the activity of *RHBDL2* has been identified in many tumour cells including breast cancer [35]. The role of *FOXC1* as a regulator of human breast cancer cells by activating NF *κ*B signaling has been discovered in a recent work [36].

#### TFBS enrichment analysis

To analyse the potential coregulation of coexpressed genes, we have done transcription factor binding site (TFBS) enrichment analysis using the TRANSFAC library (version 2009.4) [37] that contains eukaryotic transcription factors, their experimentally proven binding sites, and regulated genes. Here we used 42,544,964 TFBS predictions that have high affinity scores and are conserved between human, mouse, dog and cow [38]. Out of these 42 million conserved TFBSs we have selected the best 1% for each TRANSFAC matrix individually to identify the most specific regulator (transcription factor) - target interactions. We have used hypergeometric test [39] and Benjamini Yekutieli-FDR method [40] for p-value correction to find over-represented binding sites (p-value ≤ 0.05) in the upstream regions of genes belonging to each tricluster. Table 3 shows the list of triclusters where we have found statistically meliorated TFBSs. From Table 3, we can observe that the genes in tricluster 26 are enriched with helix-turn-helix, zinc-coordinating DNA-binding and basic domain transcription factors. The helix-turn-helix domain transcription factor E2F1, to which TRANSFAC matrix V$E2F_Q2 is associated acts as a regulator of cell proliferation in estrogen-induced breast cancer cell [41]. The zinc finger transcription factors Sp1 and Sp4, asociated with matrix V$SP1_Q6_01 have already been reported to play an important role in estrogen-induced MCF-7 breast cancer cell line [42,43]. In tricluster 17, the basic domain transcrption factor CREB (matrix V$CREB_01) is important for malignancy in breast cancer cell. ATF1, ATF2, ATF3, ATF4, ATF5 (matrix V$CREBATF_Q6) likewise play an important role in breast cancer cell [44]. We have observed enrichment for matrix V$NFAT1_Q6. The corresponding transcription factor (NFATC1) has been found to be associated with clinical characteristics in breast cancer cell [45]. In tricluster 4 POU2F1, the TF associated with matrix V$OCT1_03 is a helix-turn-helix domain transcription factor (Oct-1) and has been reported to be estrogen-responsive in a previous study [46]. Table 4 shows some statistically enriched KEGG pathway terms for coexpressed and differentially expressed (using adjusted p-value ≤ 0.05) genes the promoters of which are bound by aforementioned transcription factors.

**Table 3:** TRANSFAC Matrices for Triclusters, having statistically enriched TFBS for real-life dataset

**Tricluster (no. of genes)**	**20 most significant TRANSFAC matrices (in ascending order of p-values)**		**FDR-BY corrected p-value of top-most matrix**
Tricluster 3 (875)	V$NCX_02, V$MSX1_02, V$PAX4_02, V$POU3F2_01, V$TBP_01, V$BRN3C_01, V$BARX2_01, V$HB24_02, V$HOXD10_01, V$BARX1_01, V$DBX1_01, V$HMBOX1_01, V$HDX_01, V$BSX_01, V$NKX52_01, V$HMX3_02, V$LBX2_01, V$HOXD13_01, V$NFAT1_Q6, V$HOXD8_01		4.29e-08
Tricluster 1 (4477)	V$NCX_02, V$HDX_01, V$BCL6_01, V$ZNF333_01, V$DLX2_01, V$DLX7_01, V$DLX5_01, V$SRY_02, V$BARX1_01, V$SOX4_01, V$NKX24_01, V$HOXD3_01, V$LBX2_01, V$LHX61_02, V$SRY_01, V$TST1_01, V$DLX3_01, V$XVENT1_01, V$EVX1_01, V$BARX2_01		1.27e-05
Tricluster 26 (3177)	V$E2F_Q2, V$ZF5_01,V$USF2_Q6, V$SP1_Q6_01, V$KID3_01, V$CHCH_01		2.99e-05
Tricluster 4 (3482)	V$BCL6_01, V$HOXA10_01, V$SRY_01, V$NKX23_01, V$WT1_Q6, V$HOXB9_01, V$ISL2_01, V$HOXD10_01, V$HOXD8_01, V$NCX_02, V$X1_02, V$PAX4_04, V$BARHL2_01, V$DLX1_01, V$SRY_02, V$OCT1_03, V$DLX5_01, V$LHX9_01, V$DBX2_01, V$HMGIY_Q6		9.51e-05
Tricluster 2 (2186)	V$CHCH_01, V$MOVOB_01, V$MAZ_Q6, V$PAX4_03, V$CACD_01, V$GEN_INI3B_B, V$GEN_INI_B, V$CKROX_Q2		0.0001
Tricluster 12 (476)	V$SRY_02, V$NCX_02, V$BCL6_01, V$HB24_01, V$HOXA10_01, V$NKX25_02, V$SRY_01, V$PBX1_02, V$HOXD10_01		0.002
Tricluster 17 (999)	V$CREB_01, V$CREBATF_Q6, V$SP1_Q6_01, V$ATF3_Q6, V$CREBP1CJUN_01		0.004
Tricluster 50 (182)	V$ETF_Q6		0.006
Tricluster 18 (260)	V$STAT1STAT1_Q3		0.042
Tricluster 31 (2465)	V$SP1_Q6_01		0.046

**Table 4:** Statistically enriched KEGG pathway terms for differentially expressed and coexpressed targets of TRANSFAC matrices V$NFAT1_Q6, V$OCT1_03, V$CREB_01, V$CREBATF_Q6, V$E2F_Q2 and V$SP1_Q6_01

**Tricluster**	**TRANSFAC matrix**	**KEGG pathway terms (corrected p-value ≤ 0.05)**
4	V$NFAT1_Q6	KEGG: 00471: D-Glutamine and D-glutamate metabolism (*GLS*), KEGG: 04310: Wnt signaling pathway (*PPP2R1B, ROCK1, TBL1X*), KEGG: 04350: TGF-beta signaling pathway (*ROCK1, TBL1X*),
4	V$OCT1_03	KEGG: 04961: Endocrine and other factor-regulated calcium reabsorption (*SLC8A1, ESR1*)
17	V$CREB_01	KEGG: 00030: Pentose phosphate pathway (*RBKS*), KEGG: 04012: ErbB signaling pathway (*PAK1*), KEGG: 05211: Renal cell carcinoma (*PAK1*)
17	V$CREBATF_Q6	KEGG: 04660: T cell receptor signaling pathway (*PAK1*), KEGG: 04650: Natural killer cell mediated cytotoxicity (*PAK1*, KEGG: 05120: Epithelial cell signaling in Helicobacter pylori infection (*PAK1*), KEGG: 04360: Axon guidance (*PAK1*)
26	V$E2F_Q2	KEGG: 04110: Cell cycle (*MCM7, ANAPC1, WEE1*), KEGG: 03030: DNA replication (*MCM7, POLA1*)
26	V$SP1_Q6_01	KEGG: 00100: Steroid biosynthesis (*SQLE*), KEGG: 00270: Cysteine and methionine metabolism (*MAT2A*), KEGG: 04962: Vasopressin-regulated water reabsorption (*CREB1*), KEGG: 04623: Cytosolic DNA-sensing pathway (*MAVS*)

## Conclusion

In this work we have proposed *δ*-TRIMAX triclustering algorithm that aims to retrieve large and coherent groups of genes, having an MSR score below a threshold *δ*. Genes belonging to each tricluster are coexpressed over a subset of samples/ experimental conditions and across subset of time points. The results of GO and KEGG pathway enrichment analysis show that our proposed algorithm is able to extract group of coexpressed genes that are biologically significant. We have performed TFBS enrichment analysis to establish the fact that the promoter regions of the genes having similar expression profile are bound by the same transcription factors. We have compared the performance of our algorithm with that of existing algorithm using one artificial dataset in terms of affirmation score and one real-life dataset in terms of coverage, statistical difference from background and triclustering quality index score. In case of these two datasets our proposed algorithm outperformed the existing one. Additionally, here we have represented the expression profiles of genes belonging to each tricluster by eigengene and then identified hub genes using the profile of eigengene. We have observed that most of the identified hub-genes are previously reported to be associated with breast cancer and estrogen responsive elements. The other identified hub genes might be associated with breast cancer and need to be verified experimentally. Hence our integrative approach and findings might provide new insights into breast cancer prognosis.

## Competing interests

The authors declare that they have no competing interests.

## Authors’ contributions

AB, MH, AM, UM and SB carried out the literature study and preplanning of this work. AB, MH collected the datasets. MH participated in the prediction of transcription factor binding sites. AB developed the code, did the experiments, analysed the results and wrote the draft of the manuscript. MH, AM, UM, SB and EW corrected the draft. EW supervised the entire work. All authors read and approved the final manuscript.
